# ACTIVA-Senior: Study Design and Protocol for a Preliminary Multidomain Outdoor Intervention Promoting Healthy Aging and Mitigating Psycho-Physiological Decline

**DOI:** 10.3390/healthcare13101110

**Published:** 2025-05-10

**Authors:** Antonio Manuel García-Llorente, Raquel Vaquero-Cristóbal, Antonio J. Casimiro-Andújar, J. Arturo Abraldes, Pablo J. Marcos-Pardo

**Affiliations:** 1 SPORT Research Group (CTS-1024), CIBIS (Centro de Investigación para el Bienestar y la Inclusión Social) Research Center, University of Almería, 04120 Almería, Spain; amgll1991@gmail.com (A.M.G.-L.); casimiro@ual.es (A.J.C.-A.); pjmarcos@ual.es (P.J.M.-P.); 2 Research Group Movement Sciences and Sport (MS&SPORT), Department of Physical Activity and Sport, Faculty of Sport Sciences, University of Murcia, 30720 Murcia, Spain; 3 Active Aging, Exercise and Health/HEALTHY-AGE Network, Consejo Superior de Deportes (CSD), Ministry of Culture and Sport of Spain, 28004 Madrid, Spain; 4 Department of Education, Faculty of Education Sciences, University of Almeria, 04120 Almeria, Spain

**Keywords:** outdoor, multidomain, sarcopenia, cognitive, aging, dual task, healthcare

## Abstract

The global aging trend increases chronic diseases and lowers quality of life. Exercise is vital for physiological, cognitive, and mental health, countering age-related decline. Outdoor multidomain interventions enhance adherence, motivation, and resilience, supporting independence and well-being. **Objectives**: This paper aimed to apply an outdoor exercise protocol for middle-aged and older people and to study its preliminary effects on cognitive state, body composition, cardiovascular health, physical fitness, physiological function, physical activity, frailty, incidence of sarcopenia, and satisfaction with life. **Methods**: This protocol describes an eighteen-week, two-pronged, parallel, single-blind randomized controlled trial. This paper complies with the Consort and SPIRIT guidelines. A cohort comprising a minimum of fifty-two older adults from the University for Seniors program will be equally allocated to a multidomain training group (TG) and a passive control group (CG). **Intervention**: The TG will follow a multidomain outdoor intervention twice a week for a complete duration of 18 weeks, with recommendations for additional autonomous cardiorespiratory training. The supervised sessions will be divided into a 10-min warm-up session focusing on activation and joint mobility, followed by 40 min of resistance training, cardiorespiratory training, and balance and coordination; and it concludes with a 10-min cool-down featuring flexibility, relaxation, and playful emotional intelligence tasks. Cognitive training will be integrated across different parts of the session. **Conclusions**: This preliminary study aims to explore the feasibility and potential effectiveness of outdoor multidomain training in improving the health of older adults. Importantly, by including late middle-aged adults from the age of 55, this study also aims to explore the potential of preventive strategies initiated before reaching old age. This reflects a broader conceptualization of healthy aging as a lifelong process, where early interventions may help mitigate decline and extend independence into later life. The partnership between health professionals and physical activity fosters independence for older adults, addressing the increasing burden on health services.

## 1. Introduction

The rise in life expectancy, driven by advancements in healthcare systems and other factors, has contributed to a substantial increase in the population of older adults [1,2,3]. This demographic shift brings with it a host of challenges related to physical and cognitive health [4]. As people age, they become more vulnerable to frailty, disability, and chronic diseases, leading to a decline in overall quality of life [5]. Furthermore, one of the most concerning aspects of aging is cognitive decline, which can significantly impact independence and well-being [4]. Depression also rises among middle-aged and older adults, representing a public health issue linked to emotional distress, increased risk of dementia, and higher risk of death from other causes [6,7]. This highlights the urgent need for effective interventions to prevent or delay its onset [8,9].

Although the World Health Organization (WHO) and the United Nations define older adults as individuals aged 60 years and above [2,3], emerging evidence suggests that initiating preventive interventions from late middle age—starting around 55 years old—can significantly enhance long-term health outcomes and support healthier aging trajectories [10]. This upstream approach aims to mitigate age-related decline before its onset, fostering resilience and prolonged independence. Moreover, middle-aged individuals with chronic diseases are more likely to experience a pronounced deterioration toward frailty later in life, so starting preventive work as early as possible seems like an excellent strategy to slow the effects of aging [11].

Against this background, previous research has demonstrated the positive effects of systematic physical exercise on functionality, quality of life, independence, cognitive status, and physical and psychological health in late middle-aged adults and older adults [12,13,14,15]. More specifically, cardiorespiratory exercise, such as walking or cycling, has been linked to improvements in cardiovascular fitness and cognitive function, including memory and attention [12]; while strength training, based on lifting weights or using resistance bands, can help maintain muscle mass and strength, reducing the risk of falls and frailty [13,14]. Despite these benefits, physical activity levels in the older population are exceptionally low, as evidenced by only 33% and 17% of the population aged over 65 meeting recommended cardiorespiratory and strength training thresholds by the WHO, respectively [16].

In addition to the benefits of physical exercise, cognitive training has gained attention as a potential strategy for preserving cognitive function in middle-aged and older adults [15,17,18]. Cognitive training programs typically involve exercises designed to challenge memory, attention, and problem-solving skills [19]. While the evidence for the effectiveness of cognitive training alone is mixed [20], research suggests that multidomain interventions may have synergistic effects, resulting in greater improvements compared to single-component interventions [21]. Dual-task training, combining physical exercise with cognitive tasks, may enhance cognitive flexibility by engaging executive functions, especially when the physical component is either automated or taxing enough to recruit prefrontal resources [22]. These principles are supported by recent systematic reviews and meta-analyses highlighting the superior effects of multidomain interventions over single-modality approaches [23,24]. This selection of components is grounded on the theoretical framework of “multidomain synergy”, which proposes that simultaneously targeting multiple physiological and cognitive systems produces cumulative and interactive benefits [21]. Specifically, the combination of resistance, cardiorespiratory, balance, and cognitive training is designed to enhance neuroplasticity, functional capacity, and psychosocial well-being through mechanisms such as muscle–brain crosstalk, dual-task challenge, and enriched environments [25,26,27,28].

Multidomain interventions that integrate physical and cognitive exercise have emerged as a promising approach to promoting healthy aging, reducing the risk of cognitive decline and mitigating the effects of neurodegenerative diseases [29,30,31]; improving cognitive function [17]; and improving functionality, quality of life and overall health of the older people [31,32,33,34,35,36]. Not surprisingly, previous studies have demonstrated the benefits of a two-year multidomain program in individuals aged 60–77 years old with dementia and cognitive impairment, finding an improvement or maintenance of cognitive functioning [21]. Similarly, a one-year multidomain program in individuals with pre-frailty or frailty has been shown to reduce depression, while enhancing physical, functional, and cognitive performance, thereby supporting its effectiveness in supporting healthy aging [37]. In addition, the potential benefits of multidomain exercise in mitigating physical and cognitive decline, as well as depression, have gained relevance during the COVID-19 pandemic [38,39].

These types of interventions may also hold significant potential for middle-aged adults under the age of 60, as aging is a gradual and multifaceted process that benefits from early engagement in multidomain training to promote healthy aging. Preventive strategies implemented before old age, particularly those addressing both physical and cognitive health, can foster resilience and delay age-related decline. Recent evidence has highlighted the effectiveness of these interventions even in non-older populations, supporting their role in healthy aging from an earlier stage of life [18].

It is worth noting that previous research with multi-domain interventions both in middle and older adults has mostly been carried out in indoor environments, such as fitness centers. Nonetheless, outdoor settings may offer added psychological and engagement benefits [21,29]. More specifically, outdoor exercise settings, such as purpose-built seniors’ parks or outdoor gyms, have shown promising outcomes in supporting physical health and mental well-being in older adults, beyond traditional walking-based activity. Despite logistical challenges, these environments have been found to enhance accessibility, enjoyment, and long-term engagement, suggesting that well-designed open-air spaces may serve as viable platforms for implementing multidomain programs in community settings [31].

However, there are no previous studies that have analyzed the effects of a multidomain program on healthy middle-aged and older adults with interventions of less than one year, which could help make implementation more feasible and reduce drop-out rates [25]. Furthermore, no previous research has examined the effects of a multidomain intervention conducted outdoors, even though outdoor exercise interventions are expected to become pivotal in interventions to improve the health of older people [26].

Consequently, the aim of this protocol is to conduct a preliminary study that explores the effects of an 18-week, twice-weekly outdoor multidomain intervention—including physical exercise, cognitive stimulation, and social engagement—on the health of late middle-aged and older adults. This study also seeks to assess the feasibility and acceptability of the intervention, thereby informing the design of future large-scale trials by evaluating both its potential effects and feasibility. Additionally, this study aims to assess the feasibility and acceptability of the ACTIVA intervention among older adults, to inform future larger-scale trials. We hypothesize that the ACTIVA-Senior program, conducted entirely in an open-air environment, will lead to significant improvements in cognitive, physical, and psychological outcomes. Moreover, the outdoor setting may contribute to greater adherence, enhanced social interaction, and a more enjoyable and relaxed experience, potentially amplifying the overall impact of the intervention on the participants.

This protocol describes a single-blind randomized controlled trial designed as a preliminary and exploratory study. Its dual aim is to evaluate the feasibility and acceptability of an outdoor multidomain intervention for late middle-aged and older adults and to examine its initial effects on cognitive, physical, and psychological health outcomes.

## 2. Materials and Methods

### 2.1. Design

This study intends to run an 18-week, two-pronged, parallel, single-blind randomized controlled trial (RCT; ClinicalTrial.gov NCT05481346: https://beta.clinicaltrials.gov/study/NCT05481346 (accessed on 2 August 2022)). It follows the guidelines outlined in the Consolidated Standards for Reporting Trials (CONSORT) recommendations on RCTs [40]. In addition, it adheres to the Standard Protocol Items: Recommendations for Interventional Trials (SPIRIT);both checklist are available in the Supplementary Materials. The research will take place in the sports facilities of the University of Almeria, Spain, and the evaluations will be conducted at the university’s sports science laboratory. Participants have already been selected and are awaiting the commencement of the trial. Prior to the start of the program, participants will be assessed for the pre-test, and then they will proceed to a 2-week familiarization program.

To ensure participant safety during the outdoor training, a comprehensive plan will be implemented, with all instructors trained in basic first aid and emergency response protocols. In case of injury or medical emergency, participants will be referred to medical professionals, and emergency contacts will be available. Weather-related challenges will be managed with real-time weather monitoring; sessions will be rescheduled if adverse conditions (e.g., extreme heat, rain, or storms) are predicted. Participants will be informed of weather conditions and provided with guidelines on appropriate attire.

In accordance with SPIRIT Item 13, Table 1 presents the schedule of enrolment, interventions, and assessments.

### 2.2. Ethical Considerations

Throughout the expected research, the ethical principles for studies involving human subjects, as proposed in the Declaration of Helsinki and its subsequent revisions, will be strictly adhered to. Approval from the Ethics Committee of the University of Almeria was sought (register code: UALBIO2022/011). All the future participants have now received verbal and written information about the research protocols, including an informed consent document outlining the study’s procedures, objectives, risks, benefits, and confidentiality measures. Participants will be able to withdraw at any time without explanation, and a coding system will ensure confidentiality. Only research team members will access participant data, and each participant will receive a final report with their results at the study’s conclusion.

### 2.3. Sample Size and Power Calculation

The sample size was calculated using RStudio 3.15.0 software (RStudio Inc., Boston, MA, USA) prior to the recruitment of participants. Assuming a study design with a 5% Type I error rate and 75% statistical power, the calculation was informed by effect sizes reported in similar multidomain interventions. These effect sizes were drawn from studies targeting physical, cognitive, and psycho-physiological outcomes in older adults [41,42,43]. A potential 15% dropout rate has been accounted for in the final estimate [44]. This yielded a total sample size of 52 participants (26 per group). While this sample size is considered sufficient to detect meaningful changes, the relatively small cohort may increase the risk of Type II errors. Therefore, upon completion of the trial, the results will have to be interpreted with caution and regarded as preliminary until validated by studies with larger samples.

### 2.4. Participant Recruitment

Although baseline assessments and the intervention have not yet commenced, participant recruitment and randomization have already been completed. A convenience sampling method was employed to recruit middle-aged and older adults through direct communication and email outreach within the senior university program at the University of Almeria. All potential participants were contacted individually.

**Figure 1:**
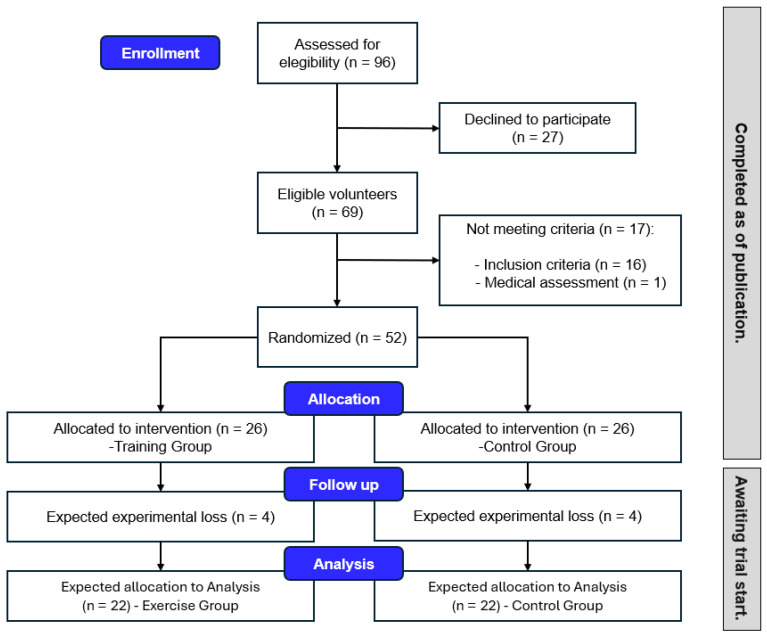
Consolidated Standards of Reporting Trials flow diagram showing the flow of participants through each stage of the study. NOTE: The follow-up accounts for potential participant exclusion based on expected attrition observed in previous studies.

To be included in the study, participants were expected to meet the following criteria: (a) aged 55 or over; (b) being physically independent; (c) being enrolled in the university for seniors’ program; and (d) passing a medical examination prior to participation, including an electrocardiogram (EKG) under stress conditions. The exclusion criteria were: (a) engaging in moderate or vigorous cardiovascular exercise systematically (≥150 min per week); (b) performing strength training systematically in the last 2 months; (c) having a body mass index (BMI) greater than 35 kg/m^2^; (d) diagnosed with ischemic heart disease, severe aortic stenosis, uncontrolled arrhythmias, decompensated heart failure, acute myocarditis, severe peripheral arterial disease, cerebrovascular accident, thromboembolic disease, aortic aneurysm, clinically significant renal or hepatic failure, severe chronic obstructive pulmonary disease (COPD), clinically significant acute infections, severe psychiatric illness, uncontrolled metabolic diseases, musculoskeletal pathologies, or osteoporosis; (e) uncontrolled blood pressure (systolic blood pressure > 180 mmHg and diastolic blood pressure > 110 mmHg); (f) other pathologies contraindicating physical exercise, identified through the mandatory medical examination that all participants underwent; (g) participants not attending both of the assessments; and (h) participants of the TG who do not attend at least 75% of the sessions (Figure 1).

### 2.5. Randomization and Blinding

Randomization was performed electronically using Microsoft Excel 2016 before the start of the program and pre/tests. An investigator not involved in participant recruitment conducted the randomization process following a randomization sequence (1:1). In addition, the examiners in charge of carrying out the evaluations will be blinded, and the participants will be instructed not to disclose the group to which they have been assigned to when the assessments are carried out.

### 2.6. Intervention

The TG will follow an outdoor multidomain training program in groups of no more than six participants. All sessions will take place in an open-air facility, equipped with sports equipment such as dumbbells, kettlebells, plyometric boxes, sandbags, resistance bands, and other functional training materials. Although cognitive training is a structured component of the intervention, activities related to emotional intelligence will be incorporated separately through playful, interactive, and socially engaging tasks. These will be intended to support emotional and relational well-being and are evaluated as an independent exploratory outcome.

The outdoor multidomain training program will include resistance training, cardiorespiratory, balance, coordination, flexibility, relaxation, playful and relational tasks related to emotional intelligence, and cognitive training. The program will be based on the HEALTHY-AGE multidomain intervention [45], and it is designed for middle-aged and older people, with the weekly distribution of session content detailed in Table 2. The program will have a duration of 18 weeks of training, with a frequency of two days per week and a duration of 60 min per session conducted face-to-face. The training sessions will be divided into three parts and will include the following components: (1) warm-up (10 min): activation exercises and joint mobility exercises; (2) main part (40 min): resistance training, cardiorespiratory training, and balance and coordination; and (3) cool-down (10 min): flexibility, relaxation, and playful and relational tasks related to emotional intelligence. Cognitive tasks will be integrated in a cross-cutting manner in all parts of the session.

The cognitive component will progressively increase in complexity across the 18-week period, beginning with basic sensorimotor and attention tasks, and gradually incorporating dual-task challenges, memory recall, and executive function elements. Trainers will be guided by a structured progression plan to ensure appropriate cognitive load and adaptation for each participant. Adherence will be monitored daily using electronic forms completed by the trainers. Attendance will be recorded for each session, along with an evaluation of each participant’s performance based on session objectives, exercise intensity, volume, and overall engagement.

In addition, to supervise multidomain training and based on the exercise recommendations [46,47], participants of the TG will be instructed to autonomously walk briskly (cardiorespiratory training) for two additional days per week, with each session lasting at least 30 min at moderate intensity, while using the talk test for perceived exertion [48]. Participants will receive training on how to use the talk test during the first week of the intervention. Additionally, participants from TG will be encouraged to use the mobile phone pedometer to keep track of the distance they cover. The intensity of the session will be monitored by the Borg Rating of Perceived Exertion Scale (target score: 6–7/10) [49]. Participants will have to keep a training diary indicating the days they complete this type of training, the time they walk, the distance covered, and the rating of perceived exertion at the end of the session, following the methodology of previous studies [45].

In every week, the same blocks of contents will be maintained for the first session of the week; and the same blocks of contents for the second session of the week. The training program was designed in accordance with the recommendations provided by the National Strength and Conditioning Association (NSCA; Fragala et al., 2019) and the American College of Sports Medicine (ACSM; Liguori, 2020), respecting the periodization to avoid reaching muscle failure and considering personalization and the nature of the effort [46,50,51].

The activation exercises will consist of different tasks and games that integrate joint mobility and some movements such as those from the main part, with a playful component while generating a progressive stimulus at the cardiorespiratory level to prepare for the session.

Resistance training will include multi-joint exercises targeting major muscle groups participating in the daily activities, including vertical and horizontal push and pull movements, hip-dominant and knee-dominant exercises, and the activation of the trunk musculature. Specific equipment such as dumbbells, barbells, kettlebells, suspension training, and resistance bands, will be used throughout the entire program [45]. The volume and intensity of the training will increase gradually on an individual basis based on ACSM and NSCA recommendations.

The resistance training in the first weekly session will consist of a circuit of local muscular endurance training (vertical organization) of 8 exercises, with 3–4 sets of 10–12 repetitions and a recovery of 20–30 between exercises and 1 between sets. As for the nature of the exercises, six of them will be strength exercises, while the other two will work on balance and coordination as the main objective. For balance and coordination work, gradual progression will be implemented by increasing and varying the base of support, support surface, type of stimulus, and information used for balance exercises. Examples will include progressing from bipodal support to a semi-tandem position, progressing from eyes open to eyes closed, and performing dynamic movements that involve the displacement of the center of gravity. Activities targeting the postural muscles such as walking on tiptoes, heels, or backwards will also be included. The intensity of the circuit will be measured by perceived exertion, aiming for an intensity of 6–7 out of 10 on the OMNI-RES Perceived Exertion Scale [49,52].

In the second session of each week, both the power training and local muscle endurance training will be included. The power block will always be carried out first and the local muscle endurance block second. For the power block, the following shall be performed: 3 exercises, with 2 sets of 6 to 8 repetitions and a recovery of 1’ between exercises and sets, following a horizontal organization. The external load will be such that a maximum of 1 or 2 repetitions in reserve (RIR) is reached, and the load will be moved as fast as possible. The intensity of this part of the session will be measured by perceived exertion, aiming for an intensity of 6–7 out of 10 on the OMNI-RES Perceived Exertion Scale [46,51].

As for the local muscle endurance training after the power work, a circuit (horizontal organization) of 3 strength exercises will be performed at a rate of 3 sets of 10 to 12 repetitions, with rests of 20 to 30’ between exercises and 1’ between sets. The intensity of this part of the session will be measured by perceived exertion, aiming for an intensity of 6–7 out of 10 on the OMNI-RES Perceived Exertion Scale [46,51].

The cardiorespiratory component of the intervention will involve two sets of four-minute bouts of continuous aerobic activity, with a 40-second rest between sets. The participants will be instructed to begin at a moderate intensity and gradually increase to a vigorous pace, guided by their ability to speak comfortably during exercise (i.e., the talk test) [48]. Exercise intensity will be further monitored using the Borg Rating of Perceived Exertion Scale, aiming for a perceived effort of 6–7 out of 10 [49].

Flexibility training will be based on passive static exercises in the cool-down period. Exercises will consist of eight stretching exercises of one or two sets of 10 to 30 s per each one, with 15 s of rest between sets and exercises. The relaxation block will consist of different breathing exercises, body–mind movements (Tai Chi, Pilates, etc.), which will integrate breathing. With respect to the emotional intelligence task, this block will consist of different games involving collaborative tasks, expression of feelings, etc.

Furthermore, cognitive training will be systematically integrated into each session through targeted, structured strategies consistent with the multidomain approach. Each session will include 2 to 4 cognitive-motor tasks, depending on duration and participant capacity, ensuring regular engagement without cognitive overload. These tasks will be standardized across sessions but adapted in complexity to match individual progression. As described in neuropsychological frameworks [22], such activities are structured to stimulate various cognitive processes while allowing for scalable difficulty. Strategies will include association tasks (e.g., linking specific movements to auditory or visual stimuli such as clapping when hearing a specific color name), memory recall and sequence reproduction (e.g., performing a series of steps in a set order and repeating them backward), and multisensory activities (e.g., touching textured objects while responding to verbal cues). Dual-task challenges—such as stepping in time while naming words starting with a given letter—will gradually increase in complexity to promote executive function. Additional components include body representation exercises (e.g., mirroring movements or identifying touched body parts with eyes closed), attention and observation games (e.g., responding only to specific shapes or colors in a movement sequence), verbal and numerical challenges (e.g., pairing movements with math operations), spatial-temporal tasks (e.g., navigating timed obstacle paths), problem-solving through movement (e.g., assembling patterns with body positioning), and guided relaxation with visualization to support mental recovery. These cognitively enriched physical tasks will aim to enhance participant engagement, promote adherence, and stimulate core cognitive domains such as memory, attention, and executive function—key targets of effective multidomain interventions [45,53].

The ACTIVA-Senior program was designed by a group of researchers from the University of Almeria, in collaboration with the Healthy Age—Active Aging, Exercise and Health Network of the “Consejo Superior de Deportes” (Spain), who have extensive experience in the design of similar interventions. The program will be delivered by five certified trainers with a BSc in Sport Science and an MSc in Physical Training.

To enhance replicability, the intervention will follow a clearly structured session format with predefined warm-up, main, and cool-down segments, which are consistently applied across the 18-week period. All sessions will be delivered by certified professionals with a BSc in Sport Science and an MSc in Physical Training, with standardized training prior to the intervention to ensure consistency. Intervention fidelity is expected to be monitored through trainer-completed session logs, including detailed records of attendance, content delivery, perceived exertion, and participant engagement. The progression of training volume and intensity will be guided by established criteria based on perceived exertion scales (OMNI-RES and Borg), with adaptations made individually to align with the participant’s capacity and safety guidelines.

Participants will be encouraged to incorporate the exercise program into their life routine after the intervention ends. Strategies will include providing participants with personalized exercise plans to continue at home, offering follow-up support through periodic check-ins, and promoting community or group-based exercise opportunities to foster social support and accountability. Additionally, resources and information on local fitness opportunities or programs will be provided to help participants stay active in the long term.

As for the participants in the CG, they will not be enrolled in any training program and will be instructed to continue with their regular level of physical activity, nutrition, and habits during the study. However, participants in the control group will undergo pre-test, post-test, re-test 1, and re-test 2 assessments and will complete the same questionnaires as the experimental group.

### 2.7. Instruments

The outcome measures include a wide range of tests and batteries focusing on cognitive state, body composition, sarcopenia, cardiovascular state, physical fitness, lifestyle, frailty, emotional state, satisfaction with life, and motivation [54,55,56,57,58,59,60,61,62,63,64,65,66,67,68,69,70,71,72,73,74]. The dimensions, tests, and their description are provided in Table 3.

### 2.8. Procedures

The participants will be evaluated at the Department of Sport Science within the University of Almeria by the same researchers, the week before (pre-test) and after (post-test) the 18-week training program. In addition, the participants will be assessed at 3- and 6-month post-intervention (re-test 1 and re-test 2, respectively) to examine the sustainability of any observed benefits across different outcome domains. Each participant will be required for two days of evaluations, each day for a duration of 60 min. Evaluations will be performed between 08:30 and 13:45, with the laboratory temperature normalized to 24 °C.

On the first day of assessments, the participants will be assessed in the following order: body composition and anthropometry variables, flexibility test (sit-and-reach test and back scratch test), cardiovascular state (blood pressure and resting heart rate), SPPB battery (chair stand test (CST), 4-m walk test (4MWT), and balance test), handgrip test, and 6-minute walk test. During the second day, participants will complete the following: sociodemographic questionnaire, psychosocial tests (CESD-R, PSQ, EQ-i-M20, SWL, and BREQ-3), physical activity questionnaire (IPAQ-S), Fried questionnaire, cognitive tests (VST and TMT test), 10-m walk test (10MWT), timed up and go test (TUG), and upper and lower limbs muscle strength.

### 2.9. Statistical Analysis

The qualitative data from sociodemographic questionnaires will be summarized using frequencies, while the rest of the quantitative variables will be described using the mean and SD for both the TG and CG across different assessment time points. To ensure robust statistical evaluation, Type I (alpha) error control will be prioritized, with the Bonferroni correction applied to set the threshold for statistical significance at *p* = 0.05. Group comparisons will adhere to the intention-to-treat principle, incorporating necessary adjustments for adherence to the exercise program. Missing data will be handled using multiple imputation based on fully conditional specification (FCS), performed in SPSS 25.0 using the Multiple Imputation module.

To assess baseline characteristic differences between groups, the Student’s *t*-test will be used for parametric numerical variables, the Mann–Whitney U test for non-parametric variables, and the chi-square (χ^2^) test for categorical binary variables. Changes within and between the TG and CG from baseline to follow-up assessments will be analyzed using the appropriate tests. For variables with a normal distribution, a repeated-measures analysis of variance (ANOVA) will be applied. Post hoc comparisons will utilize *t*-tests with Sidak corrections to control Type I errors. For non-parametric variables, the Friedman test will be followed by the Wilcoxon test, with the Bonferroni correction applied for multiple comparisons. Sensitivity analyses will be conducted to assess the robustness of the main results under varying assumptions, including analyses with and without imputed datasets and potential alternative modeling strategies.

All statistical analyses will be conducted using SPSS version 25.0. A *p*-value of <0.05 will indicate statistical significance, with adjustments made for multiple comparisons as described above.

### 2.10. Feasibility and Acceptability Assessment

Feasibility will be assessed using indicators such as recruitment rate, session attendance, retention rate, and adherence to the intervention protocol. Acceptability will be evaluated through a brief post-intervention questionnaire consisting of open-ended questions addressing participants’ satisfaction, perceived usefulness, and overall experience with the program.

## 3. Discussion

The objective of the current protocol will be to determine the feasibility and evaluate the effects of a preliminary outdoor multidomain intervention (ACTIVA-Senior program) for 18 weeks, twice per week, through a randomized controlled trial on cognitive state, body composition, sarcopenia, cardiovascular health, physical fitness, emotional state, frailty, satisfaction with life, and motivation in late middle-aged and older adults. According to the WHO [75], the period from 2020 to 2030 is designated as the “Decade of Healthy Aging”, with a key objective of promoting equality and equity of opportunities for individuals to enjoy the benefits of determinants and facilitators of healthy aging, regardless of age, sex, place of birth or residence, immigration status, or skill level. This project aligns with the “Health, demographic change, and well-being” challenge, as it aims to understand the effects of multimodal interventions on the health of older adults and to educate them on adopting a healthy lifestyle for improved quality of life [5,13]. Furthermore, by including individuals from the age of 55, this protocol emphasizes the preventive dimension of the intervention, recognizing that early engagement in multidomain training may help delay the onset of frailty and cognitive decline and support healthier aging trajectories.

The direct impact of the ACTIVA-Senior study is based on evidence demonstrating the benefits of physical exercise together with cognitive tasks in the prevention and improvement of chronic diseases among late middle-aged and older adults, educating them on adopting active aging and healthy habits, including regular participation in an appropriate physical exercise program [17,18,31]. The inclusion of feasibility and acceptability outcomes provides valuable insights into the practical implementation of the ACTIVA-Senior program, which are essential for refining the intervention and designing future larger trials.

In Spain, over half of the middle-aged and older population is affected by overweightness and chronic diseases [1]. Increased engagement in physical exercise and adoption of healthy habits can lead to a 10% reduction in healthcare expenses, with every euro invested in exercise potentially saving six euros in healthcare costs [76]. Moreover, establishing a holistic and sustainable program aimed at promoting community health is vital for combating dementia and reducing dependency [77]. These findings justify the need for non-pharmacological intervention projects that investigate the effects of multidomain training programs on health in middle-aged and older adults.

However, this study has some limitations. First, conducting a randomized controlled trial aimed at modifying healthy habits and physical activity presents challenges related to participant adherence, retention, and expectancy effects. The absence of an active control group, such as a group that participates in a health education program or other types of training, may influence behavioral and quality-of-life outcomes. However, the objective in establishing this no-treatment control group will be to obtain a realistic picture of how these variables evolve without any intervention, which is usually the case in this population. In future studies, it would be necessary to incorporate such components to improve engagement, better control for expectancy, and enhance interpretability. Second, while validated questionnaires and objective tests are employed, self-reported data remain susceptible to recall and social desirability biases. Third, although the sample size was informed by effect sizes from previous multidomain interventions, the relatively small cohort may limit generalizability and increase the risk of Type II errors. Finally, given the inclusion of a healthy population and the absence of cognitive screening upon recruitment, there is a potential for a ceiling effect in cognitive outcomes, which should be considered when interpreting future results. As such, findings will have to be interpreted with caution and viewed as preliminary pending confirmation in larger trials.

## 4. Conclusions

This preliminary study protocol outlines a randomized controlled trial designed to evaluate the efficacy and feasibility of a multidomain intervention, delivered entirely outdoors, which incorporates physical exercise and cognitive tasks aimed at promoting healthy aging and mitigating psycho-physiological decline. These approaches not only enhance the quality of life for older adults living independently but also have the potential to reduce healthcare costs by encouraging healthier aging in late middle-aged adults. This study’s findings are expected to shed light on a promising avenue for improving adherence among middle-aged and older adults, especially in outdoor multidomain interventions, an area that warrants further exploration.

## Figures and Tables

**Table 1:** SPIRIT schedule of enrolment, interventions, and assessments.

Study Period	Enrolment	Pre-Intervention (Pre-Test)	Familiarization (2 Weeks)	Intervention (Weeks 1–18)	Post-Intervention (Post-Test)	3-Month Follow-Up (Re-Test 1)	6-Month Follow-Up (Re-Test 2)
Informed Consent	X						
Eligibility Screening	X						
Randomization	X						
Baseline Assessments		X					
Multidomain Intervention			X	X			
Outcome Assessments					X	X	X

**Table 2:** Distribution of content between the weekly sessions.

Session Part	Content	Details	Monitoring	Cognitive Integration
Warm-up (10 min)	- Activation exercises - Joint mobility	Playful tasks and movement prep activities; progressive cardiorespiratory stimulus.	RPE (Borg), trainer supervision	Sensorimotor activities, coordination-based games
Main (40 min)	First weekly session Local muscle endurance (1) Balance and coordination	Circuit of 8 exercises (6 strength, 2 balance/coordination). 3–4 sets of 10–12 reps. 20–30 s rest between exercises. Equipment: dumbbells, bands, TRX.	OMNI-RES RPE (6–7/10), trainer diary	Dual tasks (e.g., movement + verbal/cognitive tasks)
	Second weekly session Power training + local muscle endurance (2) Cardiorespiratory	Power block: 3 exercises, 2 sets of 6–8 reps. Load moved as fast as possible. Local muscle endurance block: 3 exercises, 3 sets of 10–12 reps. Cardio: 2 × 4 min intervals, moderate to vigorous using the talk test.	OMNI-RES RPE, Borg scale, trainer diary	Dual tasks, attention/memory components
Cool-down (10 min)	- Flexibility - Relaxation - Emotional intelligence tasks	Static stretching (8 exercises, 10–30 s), body–mind exercises (Tai Chi, breathing), emotional intelligence games (collaboration, expression of feelings).	Trainer checklist	Breathing visualization, reflective and emotional tasks
Autonomous training	Brisk walking (2×/week)	30+ min per session, moderate intensity. Monitored via self-reported training diaries and talk test.	Training diary (duration, distance, RPE)	—

**Table 3:** Description of outcome measures.

	Dimension	Test	Description
Primary evaluations	Sociodemographic questionnaire	Ad hoc	An ad hoc questionnaire used in previous research [1] will be used to collect data on age, sex, physical independence, history of chronic diseases, and systematic exercise.
	Cognitive state	Victoria Stroop Test (VST)	The Stroop test assesses attention, inhibition, and processing speed, observing the Stroop effect where reading interferes with color identification. The test involves three phases and aims to distinguish between colors and words accurately without errors [2].
		Trail Making Test (TMT)	TMT test assesses thought flexibility and visuospatial ability. It involves connecting numbers and words in a numerical sequence without making errors.
	Body composition and anthropometric variables	Bioimpedance + Anthropometry variables + Proportionality variables	Regarding body composition, fat mass, fat-free mass, lean mass, and muscle mass will be assessed with Inbody 120. The measurements shall be carried out according to the manufacturer’s standardization conditions and previous studies [3]. Body mass, with Inbody 120; height with SECA 217 scale; and waist and hips with Lufkin tape; will be assessed according to the guidelines of the International Society for the Advancement of Anthropometry (ISAK) [4]. Subsequently, BMI (kg/m^2^), as well as waist/hip and waist/height ratio, will be calculated [5]
	Sarcopenia	Handgrip test and chair stand test (CST) (5-times sit-to-stand)	The probability of sarcopenia will be assessed using the handgrip test with a Takei tkk5401 dynamometer (P & A Medical Ltd., Duxbury, UK) and CST (5 times sit-to-stand), following the indications of the European consensus on definition and diagnosis [6]. The tests will be performed according to the protocol of previous research [7].
		Muscle mass	For the diagnosis of sarcopenia, muscle mass will be assessed using Inbody 120, following the indications of the European consensus on definition and diagnosis [6]. The tests will be performed according to the protocol of previous research [3].
		Timed Up and Go test (TUG) + Gait speed test (4 m walk test—4MWT) + Short Physical Performance Battery (SPPB)	The severity of sarcopenia (functional performance) will be assessed using TUG, gait speed, and the SPPB test, following the indications of the European consensus on definition and diagnosis [6]. The tests will be performed according to the protocol of previous research [7]. Photovoltaic cells from Microgate (Bolzano, Italy) will be used for TUG and 4MWT. The SPPB battery includes the balance test, gait speed test, and chair stand test. The Total SPPB score, ranging from 0 to 12, will be calculated by adding the scores from the three individual tests, following previous studies [8].
	Cardiovascular state	Blood pressure and resting heart rate	Blood pressure and resting heart rate will be measured using a calibrated and automated device (Omron M6W, Omron Healthcare Ltd. Hoffman Estates, IL, USA), following the protocol of previous studies [9]
	Physical fitness	Upper and lower limbs muscle strength	Upper and lower limb isometric muscle strength will be evaluated using the functional electromechanical dynamometer Dinasystem. For this purpose, the following exercises will be performed randomly, maintaining the isometric phase for 6 s during evaluation: bilateral rowing; bilateral push from the seated position with shoulder abduction at 45° and elbow flexion at 90°; and the sit-to-stand test. All of this will follow the methodology of previous research [10].
		Velocity	The 10 m walk test (10MWT) will be used for the assessment of velocity. Photovoltaic cells from Microgate (Bolzano, Italy) will be used for evaluation. This test will be conducted following the guidelines of previous research [11].
		Cardiorespiratory fitness	The 6-minute walk test will be used for the assessment of cardiorespiratory fitness. The distance covered, heart rate at the end, and heart rate recovery at 1 and 3 min will be recorded, following previous studies [12].
		Flexibility	Sit-and-reach test with a box (Acuflex, Psymtec, Madrid, Spain) and back scratch test will be used for the assessment of flexibility, following the protocol of previous studies [13]
Secondary evaluations	Physical activity (PA)	International Physical Activity Questionnaire (IPAQ-SF)	The short version of the IPAQ-SF for adults will be used to analyze levels of PA [14]
	Frailty	Fried questionnaire	The Fried questionnaire evaluates five criteria related to frailty: involuntary weight loss, exhaustion, weakness, slow gait speed, and low physical activity. The number of criteria met is classified as a three-level variable representing robustness (0 criteria met), pre-frailty (1 or 2 criteria met), and frailty (3 or more criteria met) [15].
	Emotional state	Centre for Epidemiological Studies Depression Scale-Revised (CESD-R)	The CESD-R is a 20-item questionnaire that measures depressive symptoms among older participants. Participants indicate the number of days on which they experienced depressive symptoms during the previous week using a standard four-point Likert scale. Higher scores indicate more depressive symptoms [16].
		Perceived Stress Questionnaire (PSQ)	The Perceived Stress Questionnaire (PSQ) is a psychological tool used to assess an individual’s subjective perception of stress in their daily life, focusing on feelings, thoughts, and reactions to stressful situations [17].
		Emotional intelligence in adults (EQ-i-M20)	The EQ-i-M20 is a 20-item questionnaire with subcategories such as intrapersonal, interpersonal, stress management, adaptability, and general mood [18].
	Satisfaction with life	Satisfaction with Life Scale (SWL)	The scale consists of four items rated on a Likert-type scale, ranging from 1 to 7, which assesses global subjective happiness [19].
	(e) Motivation	Behavioral Regulation during Exercise Questionnaire-3 (BREQ-3)	The BREQ-3 will be used to assess motivational regulation related to physical exercise. The questionnaire consists of twenty-three items distributed across three dimensions based on the Self-Determination Theory (SDT), which distinguishes between autonomous motivation, controlled motivation, and amotivation [20].

## Supplementary Materials

The following supporting information can be downloaded at: https://www.mdpi.com/article/10.3390/healthcare13101110/s1, Annex S1. Cut-off points for each tool/test. Table S1. Reporting checklist for protocol of a clinical trial: based on the SPIRIT guidelines. Reporting checklist for randomized trial: based on the CONSORT guidelines.

## Abbreviations

The following abbreviations are used in this manuscript:

RCT	Randomized controlled trial
TG	Training group
WHO	World Health Organization
CG	Control group
CONSORT	Consolidated Standards for Reporting Trials
SPIRIT	Standard Protocol Items: Recommendations for Interventional Trials
SD	Standard deviation
CI	Confidence interval
EKG	Electrocardiogram
BMI	Body mass index
COPD	Chronic obstructive pulmonary disease
NSCA	National Strength and Conditioning Association
ACSM	American College of Sports Medicine
RIR	Repetitions in reserve
10MWT	10 m walking test
TUG	Time up and go
χ2	Chi-square
ANOVA	Repeated measures analysis of variance
