# Supplementary material for: ACTIVA-Senior: Study Design and Protocol for a Preliminary Multidomain Outdoor Intervention Promoting Healthy Aging and Mitigating Psycho-Physiological Decline
Source: Healthcare (Basel). 2025 May 10;13(10):1110. doi: 10.3390/healthcare13101110 (PMC12111791; doi:10.3390/healthcare13101110)
Supplement: Supplementary file 1 [file healthcare-13-01110-s001.zip › healthcare-3563003-supplementary.pdf]

Annex S1. Cut-off points for each tool/test.

Outcome Measure	Tool / Test	Cut-off / Interpretation	Reference / Source
**Sarcopenia**	Handgrip Strength	< 27 kg (men), < 16 kg (women)	EWGSOP2 (2019)
	Chair Stand Test (5xSTS)	> 15 seconds suggests low muscle strength	EWGSOP2 (2019)
	Muscle Mass (InBody 120)	ASM < 7.0 kg/m^2 (men), < 5.5 kg/m^2 (women)	EWGSOP2 (2019)
	Gait Speed (4MWT)	< 0.8 m/s indicates poor physical performance	EWGSOP2 (2019)
	Timed Up and Go (TUG)	> 20 seconds suggests high fall risk	Podsiadlo & Richardson, 1991
	SPPB	Total score < 8 indicates mobility limitation	Guralnik et al., 1994
**Body Composition**	BMI	< 18.5 = underweight; 18.5-24.9 = normal weight; ≥ 25 = overweight; ≥ 30 = obese	WHO classification
	Waist-to-Hip Ratio	≥ 0.90 (men), ≥ 0.85 (women) = increased risk	WHO
	Waist-to-Height Ratio	> 0.50 = increased cardiometabolic risk	Ashwell et al., 2005
**Cardiorespiratory Fitness**	6-Minute Walk Test	Reference norms vary by age/sex; lower quartile scores suggest reduced endurance	Enright & Sherrill, 1998
**Strength (Upper/Lower Limb)**	Isometric Strength (Dinasystem)	No fixed cut-offs; scores compared to baseline and age-appropriate normative data	Previous studies (e.g., Suni et al.)
**Cognition**	Stroop Test (VST)	No cut-off; analyze interference score and errors; lower times and fewer errors = better performance	Golden, 1978
	Trail Making Test (TMT-A/B)	No clinical cut-offs; longer times suggest cognitive decline (normative data by age available)	Bowie & Harvey, 2006
**Flexibility**	Sit-and-Reach / Back Scratch	No cut-offs; compare to normative values by age/sex	Rikli & Jones, 1999
**Frailty**	Fried Criteria	0 = robust; 1–2 = pre-frail; ≥ 3 = frail	Fried et al., 2001

**Outcome Measure**	**Tool / Test**	**Cut-off / Interpretation**	**Reference / Source**
**Physical Activity**	IPAQ-SF	< 600 MET-min/week = insufficiently active; 600–3000 = moderate; > 3000 = high	IPAQ scoring protocol
**Emotional State**	CESD-R	Score ≥ 16 indicates risk of depression	Radloff, 1977
	PSQ	Higher scores = more perceived stress; no universal cut-off, but quartiles often used	Levenstein et al., 1993
	EQ-i-M20	No clinical cut-offs; interpretation based on dimensional subscale averages	Bar-On, 2006
**Satisfaction with Life**	SWLS	5–9 = extremely dissatisfied, 20 = neutral, 31–35 = extremely satisfied	Diener et al., 1985
**Motivation**	BREQ-3	No diagnostic cut-offs; use subscale means to determine motivation type (e.g., autonomous vs. controlled)	Markland & Tobin, 2004

TABLE S1. Reporting checklist for protocol of a clinical trial: based on the SPIRIT guidelines.

Reporting Item			Page Number
**Administrative information**			
Title	1	Descriptive title identifying the study design, population, interventions, and, if applicable, trial acronym	ACTIVA-Senior: Study Design and Protocol for a Multidomain Outdoor Intervention Promoting Healthy Aging and Mitigating Psycho-Physiological Decline (1).
Trial registration	2a	Trial identifier and registry name. If not yet registered, name of intended registry	For more information about the trial registration, please refer to the trail registration on the Title Page.
	2b	All items from the World Health Organization Trial Registration Data Set	
Protocol version	3	Date and version identifier	N/A
Funding	4	Sources and types of financial, material, and other support	For details of research funding support, please refer to the funding statement on the Title Page.
Roles and responsibilities	5a	Names, affiliations, and roles of protocol contributors	For more information about the roles and responsibilities of the protocol contributor, see author contributions on the Title Page.
	5b	Name and contact information for the trial sponsor	The name and contact information of the trial sponsor are on the funding statement before the references.
	5c	Role of study sponsor and funders, if any, in study design; collection, management, analysis, and interpretation of data; writing of the report; and the decision to submit the report for publication, including whether they will have ultimate authority over any of these activities	The study funders were not involved in the study design, and will not be involved in the data collection, data analysis, and writing for publication.
	5d	Composition, roles, and responsibilities of the coordinating center, steering committee, endpoint adjudication committee, data management team, and other individuals or groups overseeing the trial, if applicable (see Item 21a for data monitoring committee)	The data management team and other supervisory persons or groups consists of two graduate students in physical activity and sport sciences and two PhDs with a specialization in public health who are tutors to the students. There is one Neuropsychologist who will not actively participate in the study but may be consulted in the analysis of the cognitive outcomes.
**Introduction**			

Background and rationale	6a	Description of research question and justification for undertaking the trial, including summary of relevant studies (published and unpublished) examining benefits and harms for each intervention	(1-5) Abstract/ Introduction/ Background
	6b	Explanation for choice of comparators	The control group and the intervention group came from the same population. The establishment of the control group can eliminate the interference of other factors on the experimental results, and increase the credibility and acceptability of the experimental results.
Objectives	7	Specific objectives or hypotheses	(8) At the end of the introduction.
Trial design	8	Description of trial design including type of trial, allocation ratio, and framework	A blinded randomized controlled trial
**Methods: Participants, interventions, and outcomes**			
Study setting	9	Description of study settings and list of countries where data will be collected. Reference to where list of study sites can be obtained	(8) 2.1 Design
Eligibility criteria	10	Inclusion and exclusion criteria for participants. If applicable, eligibility criteria for study centers and individuals who will perform the interventions	(9-11) 2.4 Participant recruitment
Interventions	11a	Interventions for each group with sufficient detail to allow replication, including how and when they will be administered	(11) 2.6 Intervention
	11b	Criteria for discontinuing or modifying allocated interventions for a given trial participant	Since the current intervention is a nonpharmacological intervention, it is unlikely that adverse effects will occur due to the intervention.
	11c	Strategies to improve adherence to intervention protocols, and any procedures for monitoring adherence	(11) 2.6 Intervention
	11d	Relevant concomitant care and interventions that are permitted or prohibited during the trial	Patients will not be allowed to receive other psychological interventions during the trial.
Outcomes	12	Primary, secondary, and other outcomes, including the specific measurement variable, analysis metric, method of aggregation, and time point for each outcome. Explanation of the clinical relevance of chosen efficacy and harm outcomes is strongly recommended	(14-19) 2.8 Procedures
Participant timeline	13	Time schedule of enrolment, interventions (including any run-ins and washouts), assessments, and visits for	Details of the participant timeline are shown in Figure 1.

		participants. A schematic diagram is highly recommended (see Figure)	
Sample size	14	Estimated number of participants needed to achieve study objectives and how it was determined, including clinical and statistical assumptions supporting any sample size calculations	(10) 2.3 Sample size and power calculation
Recruitment	15	Strategies for achieving adequate participant enrolment to reach target sample size	(9) 2.4 Participant recruitment
**Methods: Assignment of interventions (for controlled trials)**			
Allocation sequence generation	16a	Method of generating the allocation sequence, and list of any factors for stratification. To reduce predictability of a random sequence, details of any planned restriction should be provided in a separate document that is unavailable to those who enroll participants or assign interventions	(11) 2.5 Randomization and blinding, participants were assigned to two groups
Allocation concealment mechanism	16b	Mechanism of implementing the allocation sequence, describing any steps to conceal the sequence until interventions are assigned	(11) 2.5 Randomization and blinding, participants were assigned to two groups
Allocation implementation	16c	Who will generate the allocation sequence, who will enroll participants, and who will assign participants to interventions	(11) 2.5 Randomization and blinding, participants were assigned to two groups
Blinding	17a	Who will be blinded after assignment to interventions, and how	(11) 2.5 Randomization and blinding, participants were assigned to two groups
	17b	If blinded, circumstances under which unblinding is permissible, and procedure for revealing a participant's allocated intervention during the trial	(11) 2.5 Randomization and blinding, participants were assigned to two groups
**Methods: Data collection, management, and analysis**			
Data collection methods	18a	Plans for assessment and collection of outcome, baseline, and other trial data, including any related processes to promote data quality and a description of study instruments along with their reliability and validity, if known. Reference to where data collection forms can be found, if not in the protocol	(15) 2.8 Procedures
	18b	Plans to promote participant retention and complete follow-up, including list of any outcome data to be collected for participants who discontinue or deviate from intervention protocols	The trainer will motivate participants during the program. Participants who drop out or deviate from the intervention protocols will not be assessed for outcomes.

Data management	19	Plans for data entry, coding, security, and storage, including any related processes to promote data quality. Reference to where details of data management procedures can be found, if not in the protocol	(20) 2.9 Statistical analysis
Statistics methods	20a	Statistical methods for analyzing primary and secondary outcomes. Reference to where other details of the statistical analysis plan can be found, if not in the protocol	(20) 2.9 Statistical analysis
	20b	Methods for any additional analyses	n/a
	20c	Definition of analysis population relating to protocol non-adherence, and any statistical methods to handle missing data	(20) 2.9 Statistical analysis
**Methods: Monitoring**			
Data monitoring	21a	Composition of data monitoring committee (DMC); summary of its role and reporting structure; statement of whether it is independent from the sponsor and competing interests; and reference to where further details about its charter can be found, if not in the protocol. Alternatively, an explanation of why a DMC is not needed	(22) 2.9 Statistical analysis
	21b	Description of any interim analyses and stopping guidelines, including who will have access to these interim results and make the final decision to terminate the trial	The intervention will be terminated if the participant indicates she/he no longer wishes to participate.
Harms	22	Plans for collecting, assessing, reporting, and managing solicited and spontaneously reported adverse events and other unintended effects of trial interventions or trial conduct	The whole procedure will strictly follow the principles of voluntariness, confidentiality, and non-harm.
Auditing	23	Frequency and procedures for auditing trial conduct, if any, and whether the process will be independent from investigators and the sponsor	Frequency and procedures for auditing trial conduct will mainly be controlled by the corresponding author and the implementor of the intervention.
**Ethics and dissemination**			
Research ethics approval	24	Plans for seeking research ethics committee/ institutional review board (REC / IRB) approval	(9) 2.2 Ethical considerations<br><br>This study protocol was approved by the University Ethics Committee (code n. UALBIO2022/011) with the consent and support of the relevant hospital departments.

Protocol amendments	25	Plans for communicating important protocol modifications to relevant parties	N/A
Consent or assent	26a	Who will obtain informed consent or assent from potential trial participants or authorized surrogates, and how (see Item 32)	(9) 2.4 Participant recruitment
	26b	Additional consent provisions for collection and use of participant data and biological specimens in ancillary studies, if applicable	No biological specimens will be collected from subjects in this intervention as ancillary studies.
Confidentiality	27	How personal information about potential and enrolled participants will be collected, shared, and maintained in order to protect confidentiality before, during, and after the trial	The data that support the findings of this study are available on request from the corresponding author. The data are not publicly available due to privacy or ethical restrictions.
Declaration of interests	28	Financial and other competing interests for principal investigators for the overall trial and each study site	No conflict of interest was declared by the authors.
Data access	29	Statement of who will have access to the final trial dataset, and disclosure of contractual agreements that limit such access for investigators	The data that supports the findings of this study are available on request from the corresponding author.
Ancillary and post-trial care	30	Provisions, if any, for ancillary and post-trial care, and for compensation to those who suffer harm from trial participation	It is unlikely that adverse effects will occur due to the intervention.
Dissemination policy	31a	Plans for investigators and sponsor to communicate trial results to participants, healthcare professionals, the public, and other relevant groups, including any publication restrictions	The results databases will be published as a paper.
	31b	Authorship eligibility guidelines and any intended use of professional writers	Authorship eligibility guidelines will be used by professional writers.
	31c	Plans, if any, for granting public access to the full protocol, participant-level dataset, and statistical code	Due to privacy or ethical restrictions, personal data is not publicly available and only present in the paper as a research result.
**Appendices**			
Informed consent materials	32	Model consent form and other related documentation given to participants and authorized surrogates	This information should be obtained from the corresponding author.
Biological specimens	33	Plans for collection, laboratory evaluation, and storage of biological specimens for genetic or molecular analysis in the current trial and for future use in ancillary studies, if applicable	No biological specimens will be collected from subjects in this study.

Table S2. Reporting checklist for randomized trial: based on the CONSORT guidelines.

Reporting Item			Page Number
**Title and Abstract**			
Title	1a	Identification as a randomized trial in the title.	ACTIVA-Senior: Study Design and Protocol for a Multidomain Outdoor Intervention Promoting Healthy Aging and Mitigating Psycho-Physiological Decline.
Abstract	1b	Structured summary of trial design, methods, results, and conclusions	Page 1(Abstract)
**Introduction**			
Background and objectives	2a	Scientific background and explanation of rationale	Page 2 (1. Introduction)
	2b	Specific objectives or hypothesis	Page 3 (Last paragraph of 1. Introduction)
**Methods**			
Trial design	3a	Description of trial design (such as parallel, factorial) including allocation ratio.	Page 3 (2.1 Design)
	3b	Important changes to methods after trial commencement (such as eligibility criteria), with reasons	n/a
Participants	4a	Eligibility criteria for participants	Page 4 (2.4 Participant recruitment)
	4b	Settings and locations where the data were collected	Page 4 (2.4 Participant recruitment)
Interventions	5	The experimental and control interventions for each group with sufficient details to allow replication, including how and when they were administered	Page 5 (2.6 Intervention)
Outcomes	6a	Completely defined prespecified primary and secondary outcome measures, including how and when they were assessed	Page 8 (2.7 Instruments)
	6b	Any changes to trial outcomes after the trial commenced, with reasons	n/a

Sample size	7a	How sample size was determined.	Page 3 (2.3 Sample size and power calculation)
	7b	When applicable, explanation of any interim analyses and stopping guidelines	The intervention will be terminated if the participant indicates she/he no longer wishes to participate.
Randomization - Sequence generation	8a	Method used to generate the random allocation sequence.	Page 5 (2.5 Randomization and blinding participants)
	8b	Type of randomization; details of any restriction (such as blocking and block size)	Page 5 (2.5 Randomization and blinding participants)
Randomization - Allocation concealment mechanism	9	Mechanism used to implement the random allocation sequence (such as sequentially numbered containers), describing any steps taken to conceal the sequence until interventions were assigned	Page 5 (2.5 Randomization and blinding participants)
Randomization - Implementation	10	Who generated the allocation sequence, who enrolled participants, and who assigned participants to interventions	Page 5 (2.5 Randomization and blinding participants)
Blinding	11a	If done, who was blinded after assignment to interventions (for example, participants, care providers, those assessing outcomes) and how.	Page 5 (2.5 Randomization and blinding participants)
	11b	If relevant, description of the similarity of interventions	n/a
Statistical methods	12a	Statistical methods used to compare groups for primary and secondary outcomes	Page 10 (2.9 Statistical analysis)
	12b	Methods for additional analyses, such as subgroup analyses and adjusted analyses	n/a
**Results**			
Participant flow diagram (strongly recommended)	13a	For each group, the numbers of participants who were randomly assigned, received intended treatment, and were analyzed for the primary outcome	This study is on the design and improvement of the intervention program stage, and data will be presented here when the program is implemented.

	13b	For each group, losses and exclusions after randomization, together with reason	
Recruitment	14a	Dates defining the periods of recruitment and follow-up	
	14b	Why the trial ended or was stopped	
Baseline data	15	A table showing baseline demographic and clinical characteristics for each group	
Numbers analyzed	16	For each group, number of participants (denominator) included in each analysis and whether the analysis was by original assigned groups	
Outcomes and estimation	17a	For each primary and secondary outcome, results for each group, and the estimated effect size and its precision (such as 95% confidence interval)	
Outcomes and estimation	17b	For binary outcomes, presentation of both absolute and relative effect sizes is recommended	
Ancillary analyses	18	Results of any other analyses performed, including subgroup analyses and adjusted analyses, distinguishing pre-specified from exploratory	n/a
Harms	19	All important harms or unintended effects in each group (For specific guidance see CONSORT for harms)	The entire procedure will strictly follow the principles of voluntariness, confidentiality, and non-harm.
**Discussion**			
Limitations	20	Trial limitations, addressing sources of potential bias, imprecision, and, if relevant, multiplicity of analyses	Page 11 (Last paragraph of 3 Discussion)
Generalizability	21	Generalizability (external validity, applicability) of the trial findings	n/a
Interpretation	22	Interpretation consistent with results, balancing benefits and harms, and considering other relevant evidence	Page 11 (3 Discussion)
**Other information**			

Registration	<u>23</u>	Registration number and name of trial registry	For more information about the trial registration, please refer to the trail registration on the Title Page.
Protocol	<u>24</u>	Where the full trial protocol can be accessed, if available	The full trial protocol can be accessed from the first author and corresponding author.
Funding	<u>25</u>	Sources of funding and other support (such as supply of drugs), role of funders	For details of research funding support, please refer to the funding statement on the Title Page.
